# Comparative Assessment of Beeswax Alcohol and Coenzyme Q_10_ (CoQ_10_) to Prevent Liver Aging, Organ Damage, and Oxidative Stress in Hyperlipidemic Zebrafish Exposed to D-Galactose: A 12-Week Dietary Intervention

**DOI:** 10.3390/ph17091250

**Published:** 2024-09-23

**Authors:** Kyung-Hyun Cho, Ashutosh Bahuguna, Ji-Eun Kim, Yunki Lee, Sang Hyuk Lee

**Affiliations:** Raydel Research Institute, Medical Innovation Complex, Daegu 41061, Republic of Korea

**Keywords:** beeswax alcohol (BWA), coenzyme Q_10_ (CoQ_10_), cholesterol, galactose, aging, liver, inflammation, zebrafish

## Abstract

The current study was designed to compare in vivo efficacy between beeswax alcohol (BWA) and coenzyme Q_10_ (CoQ_10_) to treat fatty liver changes, oxidative stress, and damages in major organs of zebrafish by 12 weeks with high-cholesterol (HC) and galactose (Gal) supplementation. At week 12, the HC control and HC+Gal control groups showed 96% and 92% survivability, respectively, while co-supplementation of the 0.5% BWA and 1.0% BWA groups exhibited 96% and 100% survivability. However, co-supplementation of the 0.5% CoQ_10_ and 1.0% CoQ_10_ groups revealed the lowest survivability, around 92% and 89%, respectively. The 0.5% BWA and 1.0% BWA groups showed 21% (*p* < 0.001) and 41% (*p* < 0.001), respectively, lower total cholesterol (TC) than the HC+Gal control, while the 1.0% CoQ_10_ group showed only 15% lower TC than the control. Interestingly, the 0.5% BWA and 1.0% BWA groups showed 22% (*p* < 0.001) and 38% (*p* < 0.001), respectively, lower triglyceride (TG) than the HC+Gal control. However, both the 0.5% CoQ_10_ and 1.0% CoQ_10_ groups showed similar TG levels as the control, suggesting that CoQ_10_ supplementation had no effect on lowering serum TG. The 1.0% BWA group showed the highest plasma HDL-C and HDL-C/TC (%) up to 3.2-fold and 5.5-fold, respectively, higher than those of the HC+Gal control, while the 1.0% CoQ_10_ group showed 2.4-fold and 2.8-fold higher plasma HDL-C and HDL-C/TC (%), respectively, than the control. The plasma aspartate transaminase (AST) and alanine transaminase (ALT) levels were lowest in the 1.0% BWA group, 51% and 72%, respectively, lower than HC+Gal control, suggesting the lowest extent of hepatic damage. In hepatic tissue, neutrophil infiltration and interleukin (IL)-6 production were the lowest in the 1.0% BWA group, around 67% and 85%, respectively, lower than the HC+Gal control. Fatty liver change, cellular apoptosis, and cell senescence in hepatic tissue were remarkably lowered in the 1.0% BWA group, while the CoQ_10_ group showed much less effect than the BWA group. In kidney, ovary, and testis tissue, the 1.0% BWA group showed the lowest production of reactive oxygen species, the extent of cellular senescence, and cellular apoptosis with the healthiest cell morphology. In conclusion, supplementation of BWA remarkably protected the liver, kidney, ovary, and testis from oxidative damage by cholesterol and galactose consumption, with the least serum AST and ALT levels, inflammatory parameters, and senescence markers.

## 1. Introduction

Beeswax is a natural product produced by different species of honeybees [1,2]. The most commercially used beeswax is produced by the honeybee species *Apis mellifera* and *Apis cerana* [1]. Typically, beeswax is a complex mixture of more than 300 constituents that majorly comprises esters (67%), hydrocarbons of C27–C33 (12–16%), and free fatty acids (12%) [1,3]. The composition of beeswax may vary based on the families and breed of the honeybees [1]. Beeswax is processed by various methods to obtain the beeswax alcohol (BWA) [4], a typical mixture of long-chain aliphatic alcohols [1,3,5]. Nevertheless, the composition of the long-chain aliphatic alcohols (LCAAs) in BWA varies based on the beeswax source used for the BWA extraction.

BWA is well known for its antioxidant and anti-inflammatory activity [6,7,8]. BWA’s potent antioxidant, hepatoprotective, and gastroprotective role in preventing oxidation of protein and lipids in the cell membrane has been described [9,10,11]. In addition, BWA’s role in preventing LDL oxidation [12] and activity enhancement of HDL-associated PON-1 [12] and inhibition of HDL glycation has been recognized recently [13]. Despite the several beneficial effects, the most noteworthy effect of BWA was observed in protecting gastric health by improving gastric mucosa quality and quantity [11,14]. BWA oral supplementation in rats prevents gastric ulceration by augmenting antioxidant status, reducing neutrophil infiltration, and enhancing secretion and quality improvement of gastric mucosa [5]. Also, the BWA impact on preventing gastric ulcers by the inhibition of protein oxidation and generation of hydroxyl radicals along with the activity enhancement of catalase, superoxide dismutase (SOD), and glutathione peroxidase (GSH-PXs) has been depicted in the gastric mucosa strengthening its strong antioxidant property [5,11]. The antioxidant effect of BWA is not only limited to the mucosa, but it also reduces lipid peroxidation and protein oxidation and raises the activities of cellular antioxidants in the liver and brain [5,6]. In a clinical study, eight weeks of supplementation of BWA (100 mg/day) improved gastrointestinal health by reducing acidity, bloating, regurgitation, sucking, and flatulence scores [5]. A six-month BWA consumption study displayed substantial antioxidant and gastroprotective effects in middle-aged and elderly human subjects [15]. Besides the gastroprotective role, BWA also displayed a substantial effect on joint health by markedly reducing proinflammatory leukotriene B4 (LTB4) levels and the inhibition of cyclooxygenase (COX) and 5 lipoxygenase (5-LOX) activities [5]. Due to the notable advantages of BWA, it has recently been approved by the Korean Food and Drug Administration (KFDA) as a functional food ingredient to support gastric and joint health [16].

Among lipid-soluble antioxidants, coenzyme Q_10_ (CoQ_10_) displayed substantial hepatoprotective effects and prevented nonalcoholic fatty liver diseases (NAFLDs) in experimental animals and humans [17,18]. However, a recent meta-analysis of randomized controlled trials revealed a statistically non-significant decrease in lipid profiles and liver enzymes of NAFLD patients after treatment with CoQ_10_ [17]. Under a cholesterol-rich diet, CoQ_10_ supplementation improved metabolic parameters and hepatic functions in rats in the presence of high doses of atorvastatin [19]. Also, CoQ_10_, due to its antioxidant effect, prevents atorvastatin-induced myotoxicity in zebrafish embryos [20].

Aging in multi-organ systems, including the liver, kidney, and brain, is associated with functional loss and morphological changes, including a decrease in mitochondrial numbers and a reduction in hepatocyte telomere length due to the accumulation of oxidative stress and inflammation [21]. D-galactose is well known to accelerate aging via the accumulation of galactitol and H_2_O_2_, which are directly linked with the impairment of cell redox homeostasis and increased levels of inflammatory markers [22,23]. The galactose supplementation induced aging in the liver, kidney, brain, and reproductive systems with an elevation of malondialdehyde (MDA), reactive oxygen species (ROS), and proinflammatory cytokines, such as tumor necrosis factor (TNF)-alpha and interleukin (IL)-6 [24]. Moreover, D-galactose-induced aging is associated with fatty liver disease and dyslipidemia characterized by high serum cholesterol and triglyceride with low HDL-C levels [25]. Although D-galactose has been used to induce oxidative stress and to develop an aging process in rats and mice [26], there has been no study to induce artificial aging in adult zebrafish by galactose supplementation. Knowing this, in the present study, D-galactose was used as an inducer of aging in adult zebrafish, enabling the assessment of BWA and CoQ_10_ against D-galactose-induced adversity.

In our previous study, a substantial effect of BWA to prevent LDL oxidation [12], functionality improvement of HDL by augmenting PON-1 activity [12], and a protective effect against ethanol-induced toxicity in zebrafish were reported [27]. Additionally, BWA in reconstituted high-density lipoprotein (HDL) shields HDL from fructose-induced glycation and counteracts carboxymethyllysine (CML)-posed toxicity in zebrafish embryos and adults through its antioxidant and anti-inflammatory mechanisms which have been disclosed [13]. Although there is growing interest in the therapeutic potential of BWA, studies on the impact of BWA on conditions like dyslipidemia and liver toxicity, particularly induced by high cholesterol and galactose, are still scarce. To address this gap, the present study aims to evaluate the comparative effectiveness of BWA and CoQ_10_ following 12 weeks of supplementation in combating galactose-induced oxidative stress, inflammatory injuries, and dyslipidemia in hyperlipidemic zebrafish.

## 2. Results

### 2.1. Zebrafish Survivability and Body Weight

**Figure 1:**
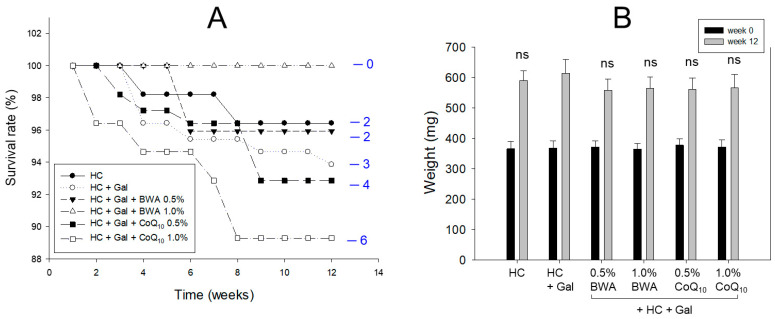
Comparative assessment of beeswax alcohol (BWA) and coenzyme Q_10_ (CoQ_10_) under a high-cholesterol (HC, final 4%, *w/w*) and galactose (Gal, final 10%, *w/w*) diet on the survivability and body weight of zebrafish. (**A**) The survival rate of zebrafish across the different groups during 12 weeks of consumption of BWA or CoQ_10_. The numerical values in the blue font represent the total number of dead zebrafish in the respective groups at 12 weeks. (**B**) Body weight of the zebrafish at the beginning (week 0) and after 12 weeks of consumption of BWA or CoQ_10_. ns indicates a non-significant difference between the groups.

During 12 weeks of consumption, as shown in Figure 1A, HC+Gal groups showed 93% survivability, while the HC alone group showed 96% survivability, suggesting that co-supplementation of galactose caused more toxic and lethal effects to zebrafish. HC+Gal+0.5% BWA and HC+Gal+1.0% BWA groups showed 96% and 100% survivability, respectively, suggesting that co-consumption of BWA prevented the HC+Gal-induced premature death. However, HC+Gal+0.5% CoQ_10_ and HC+Gal+1.0% CoQ_10_ groups exhibited 93% and 89% survivability, respectively, suggesting that co-consumption of CoQ_10_ did not prevent premature death by hyperlipidemia and galactose toxicity.

As shown in Figure 1B, during the 12 weeks, consumption of HC alone, HC+Gal, and HC+Gal with BWA or CoQ_10_ showed a nearly similar ~1.5-fold body weight enhancement with respect to the body weight observed on day 0. There is a non-significant difference in the net body weight attained at 12 weeks of consumption of different diets. The net body weight change during the 12 weeks of consumption across all the groups was in the range of 148–160% (Table 1).

### 2.2. Change in Lipid Profiles

**Figure 2:**
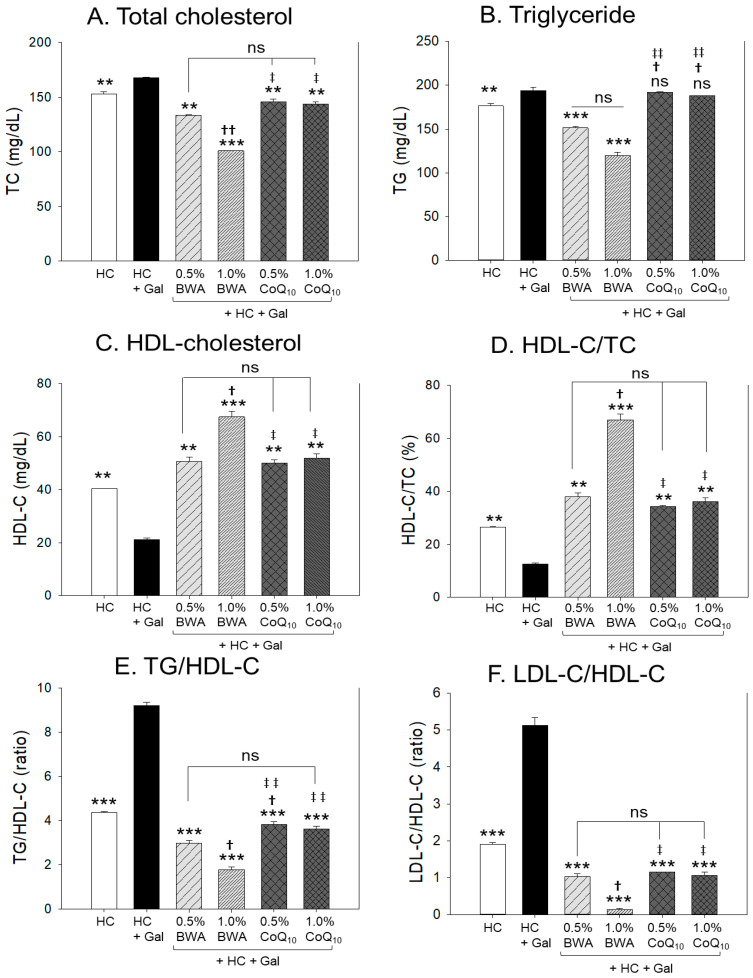
Change in plasma lipid profiles among beeswax alcohol (BWA) and coenzyme Q_10_ (CoQ_10_) groups after 12 weeks of consumption under a high-cholesterol (HC, final 4%, *w/w*) and galactose (Gal, final 10%, *w/w*) diet. (**A**) Total cholesterol (TC); (**B**) triglyceride (TG); (**C**) high-density lipoprotein cholesterol (HDL-C); (**D**) percentage of HDL-C in total cholesterol (HDL-C/TC, %); (**E**) triglycerides (TG) and HDL-C ratio; (**F**) low-density lipoprotein cholesterol (LDL-C) and HDL-C ratio; Gal, galactose; high-cholesterol diet (HC); beeswax alcohol (BWA); and coenzyme Q_10_ (CoQ_10_). The statistical divergence between the groups was denoted by ** (*p* < 0.01) and *** (*p* < 0.001) for the HC+Gal group. ^†^ (*p* < 0.05) and ^††^ (*p* < 0.01) displayed significant difference correspond to the HC+Gal+0.5% BWA group while ^‡^ (*p* < 0.05) and ^‡‡^ (*p* < 0.01) displayed significant difference correspond to the HC+Gal+1.0% BWA group; ns represents a non-significant difference between the groups.

As shown in Figure 2A,B, the HC+Gal group showed a significant 9% (*p* < 0.01) and 10% (*p* < 0.01) higher TC and TG level than the HC alone group, suggesting that co-consumption of galactose augments the HC-induced blood TC and TG levels to exacerbate hyperlipidemia. However, the 1.0% BWA group showed the lowest plasma TC and TG levels, around 41% and 39%, lower than the HC+Gal control group, respectively, while the BWA 0.5% group showed 21% and 22% lower TC and TG levels than the HC+Gal control group. In contrast, both the 0.5% and 1.0% CoQ_10_ group showed around 14% lower TC level (*p* < 0.01) than the HC+Gal control. However, both the 0.5% and 1.0% CoQ_10_ groups showed similar TG levels (187–191 mg/dL) as observed in the HC+Gal control group (193.9 mg/dL), suggesting that consumption of CoQ_10_ was not effective in reducing blood TG level.

Compared to the CoQ_10_ group, a significantly 30% (*p* < 0.05) lower TC was observed in the 1.0% BWA group. Also, a significantly 20% (*p* < 0.05) and 36% (*p* < 0.01) lower TG was quantified in the 0.5% BWA and 1.0% BWA group compared to the 1.0% CoQ_10_ group, demonstrating the superior efficacy of BWA over CoQ_10_ in minimizing HC+Gal-induced TC and TG levels.

As shown in Figure 2C,D, the HC+Gal group showed 48% and 54% lower HDL-C and HDL-C/TC (%), respectively, than those of the HC alone group, suggesting that co-consumption of galactose caused a significant lowering of HDL-C and the percentage of HDL-C in TC. The 1.0% BWA group showed the highest HDL-C and HDL-C/TC (%), around 3.2-fold and 5.5-fold higher than the HC+Gal group, while the 0.5% BWA group showed 2.4-fold and 3.2-fold higher HDL-C and HDL-C/TC (%) than those of the HC+Gal group.

As shown in Figure 2E, the HC+Gal group showed the highest TG/HDL-C ratio, around 9.2, while the HC alone group showed 4.3, suggesting that co-consumption of galactose induced a 2.1-fold more atherogenic lipid profile. The co-supplementation of 1.0% BWA showed the lowest TG/HDL-C ratio, around 1.7, followed by the 0.5% BWA group (around 2.9), suggesting that 0.5% BWA and 1.0% BWA co-consumption resulted in a 69% and 82% reduction in the TG/HDL-C ratio compared with the HC+Gal control. The co-supplementation of 0.5% and 1.0% CoQ_10_ showed around 3.8 and 3.6 TG/HDL-C ratios, respectively, which is nearly similar to the TG/HDL-C ratio observed in the HC group. The ratio of LDL-C/HDL-C (L:H ratio) was highest in the HC+Gal group, around 5.1, while 0.5% BWA and 1.0% BWA groups showed the lowest level, around 1.0 and 0.2, respectively. In contrast, 0.5% CoQ_10_ and 1.0% CoQ_10_ groups showed a 1.2 and 1.0 L:H ratio. The combined outcomes of the plasma lipid profile indicate that BWA exhibits significantly greater efficacy than CoQ_10_ in mitigating HC+Gal-induced dyslipidemia.

### 2.3. Change in Hepatic Damage Parameters

**Figure 3:**
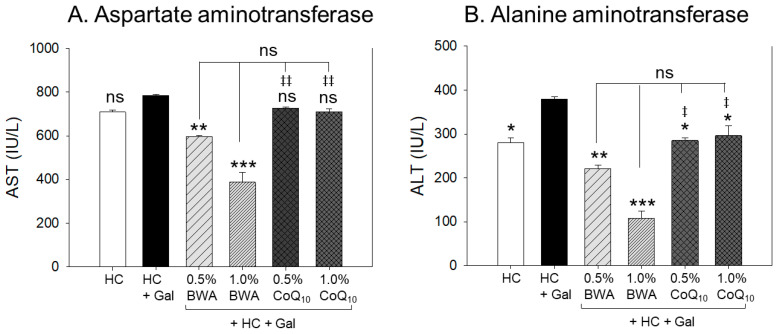
Change in plasma hepatic parameters, (**A**) aspartate aminotransferase (AST) and (**B**) alanine aminotransferase (ALT), among beeswax alcohol (BWA) and coenzyme Q_10_ (CoQ_10_) groups after 12 weeks of consumption under high-cholesterol (HC, final 4%, *w/w*) and galactose (Gal, final 10%, *w/w*) supplementation. The statistical divergence between the groups was denoted by * (*p* < 0.05), ** (*p* < 0.01), and *** (*p* < 0.001) for the HC+Gal group, while ^‡^ (*p* < 0.05) and ^‡‡^ (*p* < 0.01) correspond to the HC+Gal+1.0% BWA group; ns represents a non-significant difference between the groups.

As shown in Figure 3, the HC+Gal group showed the highest AST and ALT levels, i.e., 11% and 35% higher than the HC alone group, respectively, suggesting that co-supplementation of galactose caused more severe hepatic damage consequently increasing the AST and ALT levels. The 1.0% BWA group showed the lowest level of AST and ALT, i.e., 46% and 72%, lower than those of the HC+Gal control, respectively. The 0.5% BWA group showed the second lowest level of AST and ALT, i.e., 25% and 41%, lower than those of the HC+Gal control, respectively, suggesting the dose-dependent effect of BWA to protect HC+ Gal-induced hepatic damage. On the other hand, the 0.5% and 1.0% CoQ_10_ groups exhibited 22~25% lower ALT levels than those of the HC+Gal control; however, no significant changes in AST levels, regardless of the dosage of CoQ_10_, were observed. These results suggest that co-consumption of BWA was more effective than CoQ_10_ in preventing the hepatic damage caused by intake of HC+Gal.

### 2.4. Histologic Analysis of Liver Tissue

**Figure 4:**
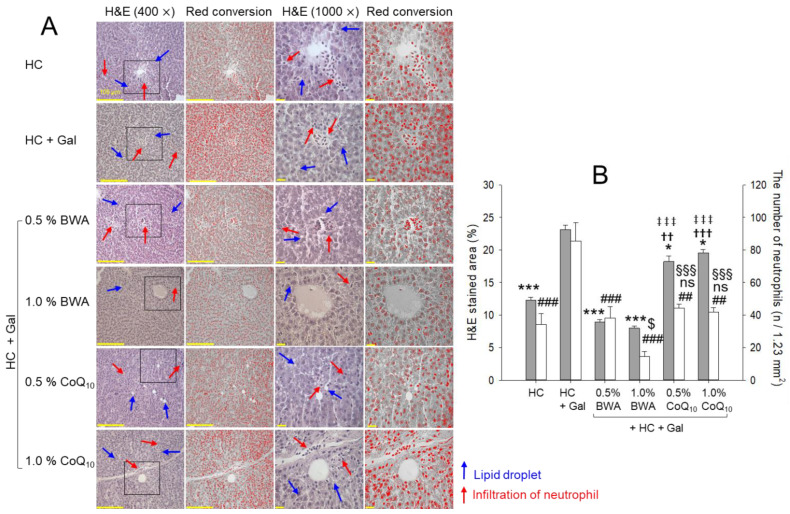
Liver histological analysis of zebrafish consuming a designated diet under high-cholesterol (HC, final 4%, *w/w*) and galactose (Gal, final 10%, *w/w*) supplementation. (**A**) Hematoxylin and eosin (H&E) staining visualized at 400× [scale bar = 100 μm] and 1000× [scale bar = 10 μm] magnification. The blue and red arrow indicates lipid droplets and infiltrated neutrophils. (**B**) Percentage of H&E-stained area and numbers of neutrophils in the H&E-stained area. A semiquantitative assessment of neutrophils (stained dark violet color) was carried out by microscopic examination of the designated area (1.23 mm^2^) across three distinct sections and five different areas of each group. The statistical divergence between the groups was denoted by * (*p* < 0.05) and *** (*p* < 0.001) for the HC+Gal group, ^††^ (*p* < 0.01) and ^†††^ (*p* < 0.001) for the HC+Gal+5% BWA group, and ^‡‡‡^ (*p* < 0.001) for the HC+Gal+1.0% BWA group for the H&E-stained area. The statistical divergence for the number of neutrophils between the groups was denoted by ^##^ (*p* < 0.01) and ^###^ (*p* < 0.001) for the HC+Gal group, ^$^ (*p* < 0.05) for the HC+Gal+0.5% BWA group, and ^§§§^ (*p* < 0.001) for the HC+Gal+1.0% BWA group; ns denotes a non-significant difference between the groups.

As shown in Figure 4A, H&E staining with a microsection of hepatic tissue revealed that the HC+Gal group showed the highest H&E-stained area and number of infiltrated neutrophils (indicated by the red arrow), which is significantly1.9-fold and 2.5-fold more than the HC group. The consumption of 1.0% BWA showed the lowest H&E-stained area and neutrophil infiltration (red arrow), which was around 67% and 83% less than that of the HC+Gal control, respectively. Additionally, the least lipid droplets (indicated by the blue arrow) were observed in the 1.0% BWA group. The 0.5% BWA group showed the second lowest H&E-stained area and neutrophil infiltration, which was around 62% and 56%, lower than the HC+Gal control, respectively. Interestingly, the 0.5% and 1.0% CoQ_10_ groups exhibited weaker efficacy towards the HC+Gal-altered H&E-stained area and neutrophil infiltration as manifested by around 16~22% and 49~52% lesser H&E-stained area and neutrophil counts than the HC+Gal group.

### 2.5. Interleukin-6 Production in Liver

**Figure 5:**
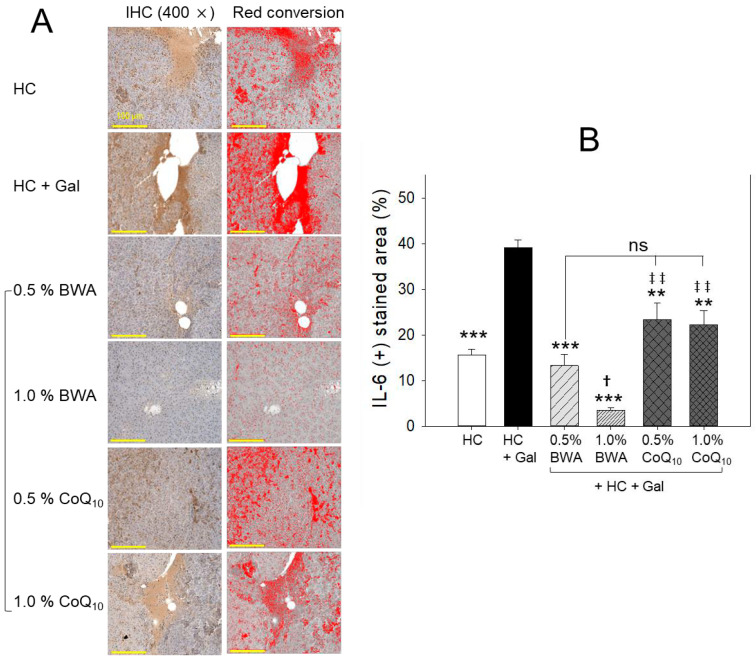
Interleukin (IL)-6 production in the liver section of zebrafish consuming a designated diet under high-cholesterol (HC, final 4%, *w/w*) and galactose (Gal, final 10%, *w/w*) supplementation. (**A**) Images of immunohistochemistry and red conversion images are IHC-stained areas (brown color) interchanged with red color [at a threshold value of (20–100)] using Image J software version 1.53r to enhance visualization. (**B**) Quantification of IL-6-stained area employing Image J software. The statistical divergence between the groups was denoted by ** (*p* < 0.01) and *** (*p* < 0.001) for the HC+Gal group and ^†^ (*p* < 0.05) for the HC+Gal+0.5% BWA group, while ^‡‡^ (*p* < 0.01) was for the HC+Gal+1% BWA group; ns represents a non-significant difference between the groups.

Immunohistochemical analysis revealed that the HC+Gal group showed the highest interleukin (IL)-6-stained area, around 39%, which was 2.5-fold higher than the HC alone group, suggesting that the co-consumption of galactose caused more hepatic inflammation (Figure 5A,B). The co-consumption of BWA remarkably decreased IL-6 production, which was around 66% and 91% lower, for the 0.5% BWA and 1.0% BWA groups, respectively, than that of the HC+Gal group. Contrary to this, the CoQ_10_ groups showed less effectiveness in minimizing the HC+Gal-induced IL-6 production. While compared to the 1.0% BWA group, a significantly ~6-fold (*p* < 0.01) higher IL-6 production was noticed in the 0.5% and 1.0% CoQ_10_-consumed groups, suggesting the lower effectiveness of CoQ_10_ than the BWA to curtail the HC+Gal-induced inflammation measured in terms of IL-6 production.

### 2.6. ROS Production, Apoptosis, Fatty Liver Change, and Cellular Senescence

**Figure 6:**
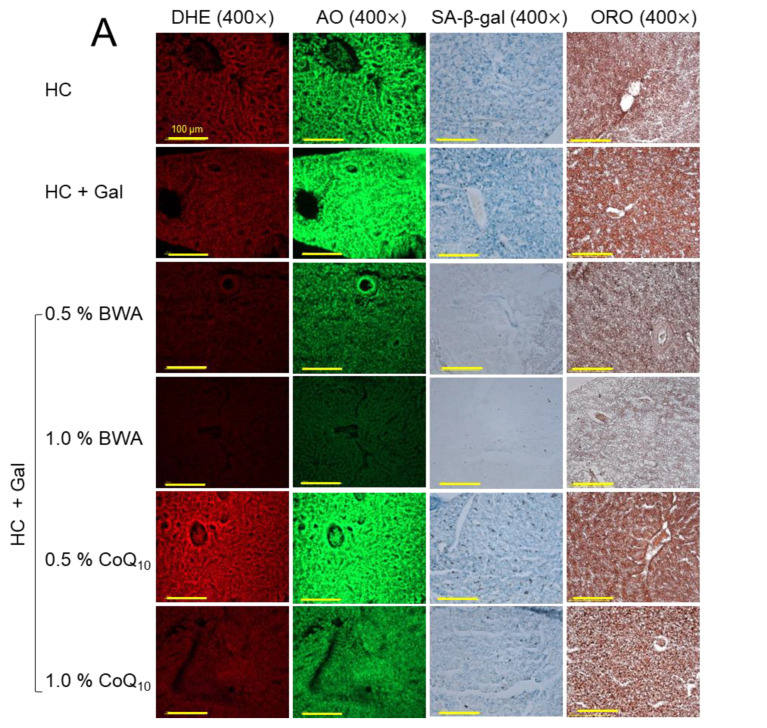

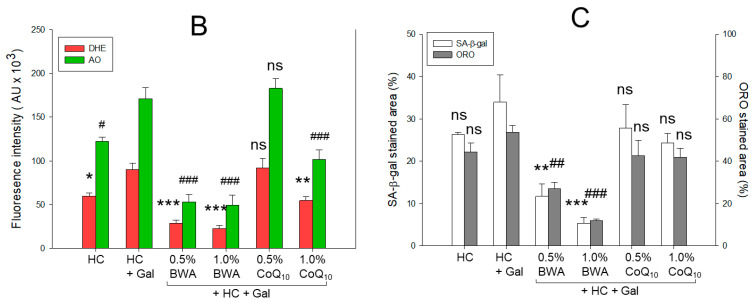
A comparative effect of beeswax alcohol (BWA) and coenzyme Q_10_ (CoQ_10_) under a high-cholesterol (HC, final 4%, *w/w*) and galactose (Gal, final 10%, *w/w*) diet on hepatic reactive oxygen species (ROS), apoptosis, senescence, and fatty liver. (**A**) Dihydroethidium (DHE) and acridine orange (AO) fluorescent staining align with reactive oxygen species (ROS) production and the extent of apoptosis, respectively. Oil red O (ORO) and senescent-associated βgalactosidase (SA-β-gal) staining. [100 μm, scale bar]. (**B**) Image J software (version 1.53r) based quantification of DHE and AO fluorescent intensity. (**C**) Quantification of ORO and SA-β-gal-stained area. The statistical divergence between the groups was denoted by * (*p* < 0.05), ** (*p* < 0.01), and *** (*p* < 0.001) for HC+Gal (for DHE and SA-β-gal) while ^#^ (*p* < 0.05), ^##^ (*p* < 0.01), and ^###^ (*p* < 0.001) were for HC+Gal (for AO and ORO); a non-significant difference between the groups is indicated by “ns”.

As shown in Figure 6A,B, the HC+Gal group showed the highest extent of ROS production (DHE-stained red fluorescence) and apoptosis (AO-stained green fluorescence), around 1.5-fold and 1.4-fold, higher than the HC alone group, respectively. The consumption of BWA effectively prevented the HC+Gal-induced ROS production, as evidenced by significantly 3.0-fold- and 3.9-fold-reduced DHE fluorescent intensity in the 0.5% and 1.0% BWA-consumed groups against the HC+Gal control group. Contrary to this, CoQ_10_ at 0.5% was found ineffective in reducing the HC+Gal-provoked ROS production; however, CoQ_10_ at 1.0% consumption significantly reduced the ROS level evident by 1.6-fold (*p* < 0.01)-reduced DHE-stained areas than the DHE-stained area appeared in the HC+Gal group. Likewise, to the outcomes of DHE fluorescent staining, the AO staining represents the significant inhibition of HC+Gal-induced apoptosis in response to the consumption of BWA at both the consumed concentrations of 0.5% and 1.0%. Contrary to this, CoQ_10_ only at 1.0% consumption displayed the antiapoptotic effect. While compared to the 0.5% and 1.0% BWA, a ~2-fold higher AO fluorescent intensity was noticed in the 1.0% CoQ_10_-consumed group, indicating higher efficacy of BWA to prevent HC+Gal-induced apoptosis in hepatic cells.

The HC+Gal group showed the highest extent of cellular senescence and fatty liver change, up to 1.3-fold and 1.2-fold, higher than the HC alone group, respectively, implying that the addition of galactose causes more severe senescence and lipid accumulation in hepatic cells (Figure 6A,C). Consumption of BWA showed a dose-dependent effect in preventing HC+Gal-induced senescence and fatty liver changes. The 1.0% BWA group showed the least extent of cellular senescence and lipid accumulation, up to 85% and 78%, less than the HC+Gal group, respectively, while compared to the 0.5% BWA-consumed groups, a ~2-fold better inhibition of cellular senescence and lipid accumulation was detected in 1.0% BWA-consumed groups. In contrast to this, CoQ_10_-consumed groups exhibited no detectable activity to improve cellular senescence and lipid accumulation (Figure 6A,C), indicating the ineffectiveness of CoQ_10_ in protecting against hepatic senescence and fatty liver damage imposed by the consumption of HC+Gal.

### 2.7. Histological Analysis of Kidney

**Figure 7:**
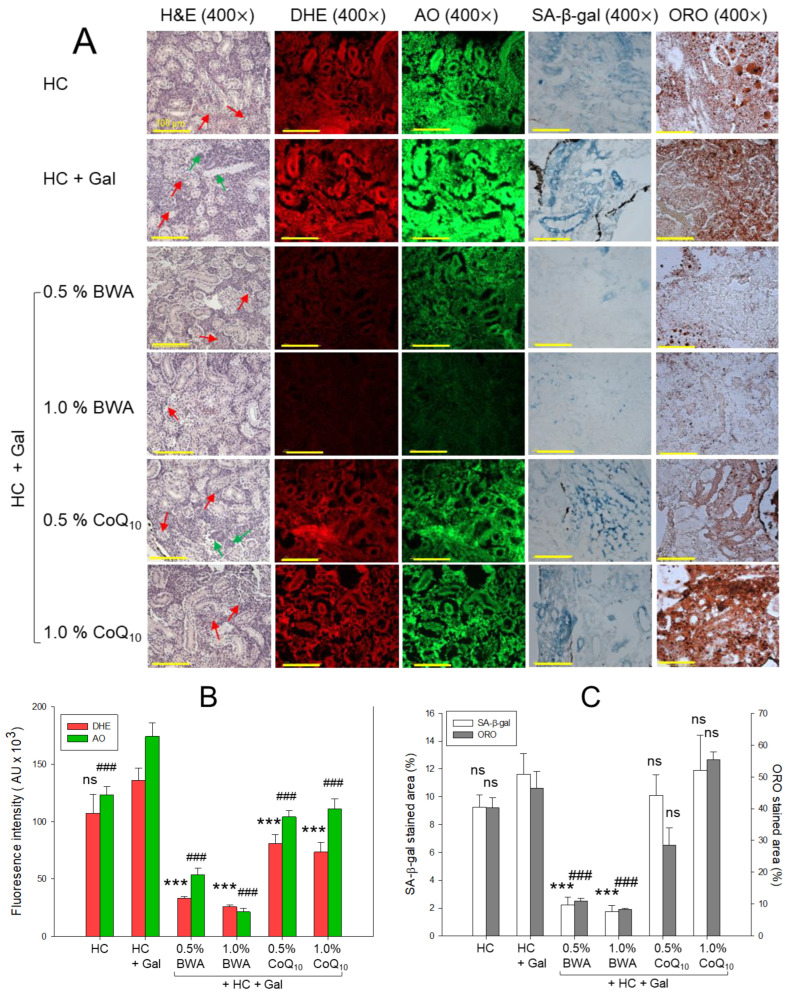
A comparative effect of beeswax alcohol (BWA) and coenzyme Q_10_ (CoQ_10_) on the kidney of zebrafish fed with a high-cholesterol (HC, final 4%, *w/w*) and galactose (Gal, final 10%, *w/w*) diet. (**A**) Hematoxylin and eosin (H&E) staining; proximal and distal tubules are abbreviated as PT and DT; and the red arrow depicts luminal debris in the tubular cast, while the green arrow depicts the basophilic cluster complying with the new nephron. Dihydroethidium (DHE) and acridine orange (AO) fluorescent staining align with reactive oxygen species (ROS) production and the extent of apoptosis, respectively. Oil red O (ORO) and senescent-associated β-galactosidase (SA-β-gal) staining. [100 μm, scale bar]. (**B**) Image J-based quantification of DHE and AO fluorescent intensity. (**C**) Quantification of ORO and SA-β-gal-stained area. The statistical divergence between the groups was denoted by *** (*p* < 0.001) for HC+Gal (for DHE and SA-β-gal) while ^###^ (*p* < 0.001) was for HC+Gal (for AO and ORO); a non-significant difference between the groups is indicated by “ns”.

The H&E staining of the kidney section highlighted the morphological changes in response to HC+Gal in the presence and absence of BWA and CoQ_10_ (Figure 7). A sparsely populated proximal and distal tubule with recurrent luminal debris in the tubular cast (indicated by a red arrow) was observed in the HC-consumed group. The consumption of Gal with HC aggravates the HC-induced kidney damage apparent by disorganized proximal tubules (PTs) and distal tubules (DTs) with frequent luminal debris and the presence of dark purple colored basophilic cluster indicating new nephron formation (highlighted by a green arrow), signifying the adverse effect of Gal in the presence of HC. The HC+Gal-induced kidney damage was considerably restored by the consumption of BWA at both the tested concentrations of 0.5% and 1.0%, evident by the well-differentiated and adequately organized proximal and distal tubules. However, the occasional luminal debris in the tubular cast was also noticed (indicated by a red arrow). Likewise, CoQ_10_ also displayed a protective effect against HC+Gal-induced kidney damage, though the efficacy is lower than the BWA. Notably, at 0.5% CoQ_10_, luminal debris with the presence of a basophilic cluster corresponding to the new nephron generation was observed; on the contrary, at 1.0% CoQ_10_, a substantial kidney preventive role was noticed.

The DHE and AO fluorescent staining documented the accentuated ROS generation and apoptosis in the HC+Gal group, which was significantly mitigated by consumption of BWA and CoQ_10_ at both the tested concentrations of 0.5% and 1.0%. A 4.1-fold- and 5.2-fold-reduced DHE fluorescent intensity was quantified in the 0.5% and 1.0% BWA-treated group that of the HC+Gal group (Figure 7A,B). Consistently, 3.2-fold- and 8.2-fold-diminished AO fluorescent intensity was noticed in 0.5% and 1.0% of the BWA-treated group compared to the HC+Gal group. Also, CoQ_10_ efficiently prevented the HC+Gal-induced ROS, as depicted by 1.6-fold- and 1.9-fold-reduced DHE fluorescent intensity in 0.5% and 1.0% CoQ_10_-treated groups, respectively. Similarly, CoQ_10_ at 0.5% and 1.0% concentrations displayed a ~1.6-fold-diminished AO fluorescent intensity with respect to the HC+Gal group. However, compared to CoQ_10_, BWA displayed a profound effect to reduce HC+Gal-induced ROS and apoptosis as quantified by 2.4-fold and 1.9-fold lower DHE fluorescent intensity and 2.6-fold- and 5.2-fold-diminished AO fluorescent intensity in the 0.5% and 1.0% BWA-consumed groups than that of the 0.5% and 1.0% CoQ_10_-consumed group, respectively.

ORO staining revealed the impact of HC+Gal on lipid accumulation in the kidney. The BWA at 0.5% and 1.0% concentrations effectively minimized HC+Gal augmented ORO-stained area by 4.2-fold and 5.6-fold compared to the HC+Gal groups (Figure 7A,C). Contrary to this, CoQ_10_ displayed no effect on preventing lipid accumulation stimulated by HC+Gal.

Similar to the ORO staining, higher cellular senescence was perceived in the HC+Gal group, which was significantly minimized by 5.2-fold and 6.7-fold by the treatment of 0.5% and 1.0% BWA, respectively (Figure 7A,C). Similar to the ORO staining, CoQ_10_ displayed no protective effect on cellular senescence actuated by the consumption of HC+Gal.

### 2.8. Examination of Ovarian Tissue

**Figure 8:**
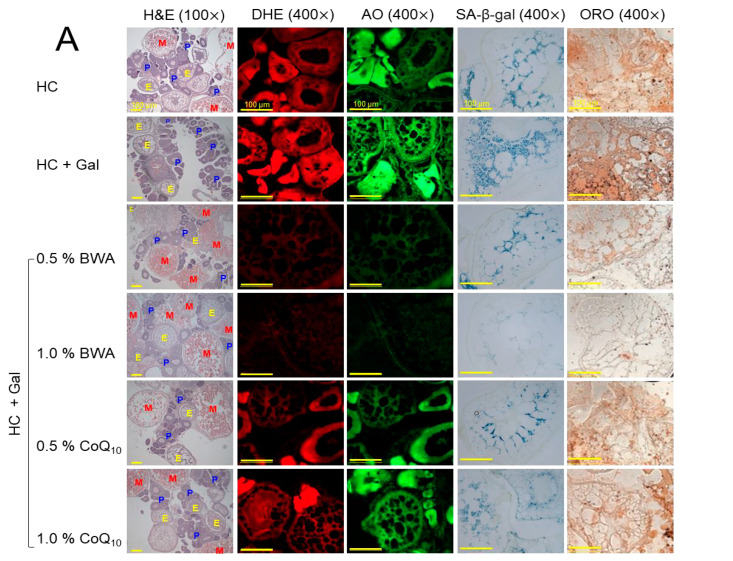

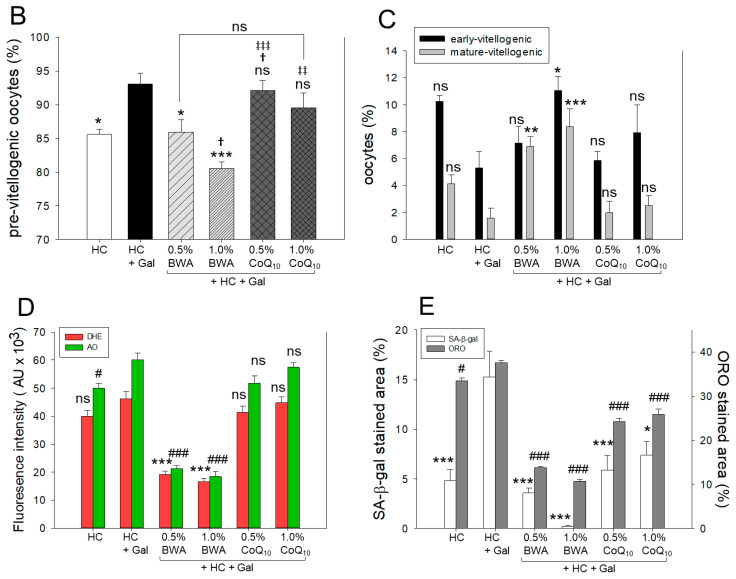
A comparative effect of beeswax alcohol (BWA) and coenzyme Q_10_ (CoQ_10_) on the ovary of zebrafish fed with a high-cholesterol (HC, final 4%, *w/w*) and galactose (Gal, final 10%, *w/w*) diet. (**A**) Hematoxylin and eosin (H&E) staining; P, E, and M highlight the pre-, early, and mature oocytes, respectively. Dihydroethidium (DHE) and acridine orange (AO) decipher the generation of reactive oxygen species (ROS) and the degree of apoptosis, respectively. Oil red O (ORO) and senescent-associated β-galactosidase (SA-β-gal) staining [100 μm, scale bar]. (**B**) Pre-vitellogenic oocyte counts. (**C**) Early and mature vitellogenic oocyte counts. (**D**) DHE and AO fluorescence intensity quantification employing Image J software. (**E**) Presence of SA-β-gal and ORO-stained areas across the different groups. The statistical divergence between the groups was denoted by * (*p* < 0.05), ** (*p* < 0.01), and *** (*p* < 0.001) for HC+Gal (for DHE fluorescent intensity and SA-β-gal-stained area) while ^#^ (*p* < 0.05), ^###^ (*p* < 0.001) were for HC+Gal (for AO fluorescent intensity and ORO-stained area). ^†^ (*p* < 0.05) was for the HC+Gal+0.5% BWA group while ^‡‡^ (*p* < 0.01) and ^‡‡‡^ (*p* < 0.001) were for the HC+Gal+1.0% BWA group; ns represents a non-significant difference between the groups.

A significantly higher prevalence of previtellogenic oocytes was noticed in the HC+Gal group, which was 8.7% higher than in the HC group (Figure 8A,B). The BWA at 0.5% and 1.0% significantly, by 7.7% and 14.1%, diminished the previtellogenic oocyte counts affected by the consumption of HC+Gal. Similarly, the HC+Gal-affected early and mature vitellogenic oocyte counts were restored considerably by BWA consumption. Precisely, BWA at a 1.0% concentration displayed significantly 2.2-fold and 5.5-fold higher early and mature oocyte counts compared to the HC+Gal group, signifying the preventive effect of BWA against HC+Gal-induced ovary impairment. Contrary to the BWA, CoQ_10_ had no impact on the ovary morphology (concerning the restoration of pre-, early, and mature vitellogenic oocytes) altered by HC+Gal consumption.

DHE staining revealed a significant dose-dependent effect of BWA in preventing HC+Gal-induced ROS generation. In response to 0.5% and 1.0% BWA consumption, a 2.4-fold- and 2.8-fold-reduced DHE fluorescent intensity was quantified in the testis section compared to the HC+Gal group. Unlike BWA, a non-significant effect of CoQ_10_ (at 0.5% and 1.0% concentrations) was noticed on the HC+Gal-induced ROS level (Figure 8A,D).

The AO fluorescent staining revealed a significantly 2.8-fold- and 3.2-fold-reduced AO fluorescent intensity in the 0.5% and 1.0% BWA-consumed group, contrary to the HC+Gal group, documenting the antiapoptotic effect of BWA (Figure 8A,D). In contrast, C_O_Q_10_ at a 0.5% concentration has a non-significant impact against HC+Gal-induced apoptosis.

A significantly 4.3-fold- and 69-fold-reduced cellular senescence was observed in the 0.5% and 1.0% BWA-consumed group compared to the HC+Gal group (Figure 8A,E). Like BWA, CoQ_10_ prevents the HC+Gal-induced cellular senescence, evidenced by ~2-fold lower SA-β-gal-positive cells in CoQ_10_ in the 0.5% and 1.0% consumption groups than that of the HC+Gal group, while compared to 0.5% and 1.0% CoQ_10_, significantly 1.6-fold and 32-fold lower SA-β gal-positive cells were detected in 0.5% and 1.0% BWA-consumed groups, signifying the functional superiority of BWA over CoQ_10_ to eliminate HC+Gal-induced cellular senescence.

The ORO staining revealed a significantly 12.2% higher lipid accumulation in the HC+BWA group than the HC group, suggesting the aggravative effect of Gal in the combination of HC towards the lipid accumulation in the ovary (Figure 8A,E). The HC+Gal-induced lipid accumulation is significantly alleviated by the consumption of BWA and CoQ_10_ as depicted by a significantly 2.7-fold-, 3.6-fold-,1.5-fold-, and 1.4-fold-reduced ORO-stained area in response to 0.5% and 1.0% BWA and CoQ_10_, respectively, against the HC+Gal group.

### 2.9. Analysis of Testicular Tissue

**Figure 9:**
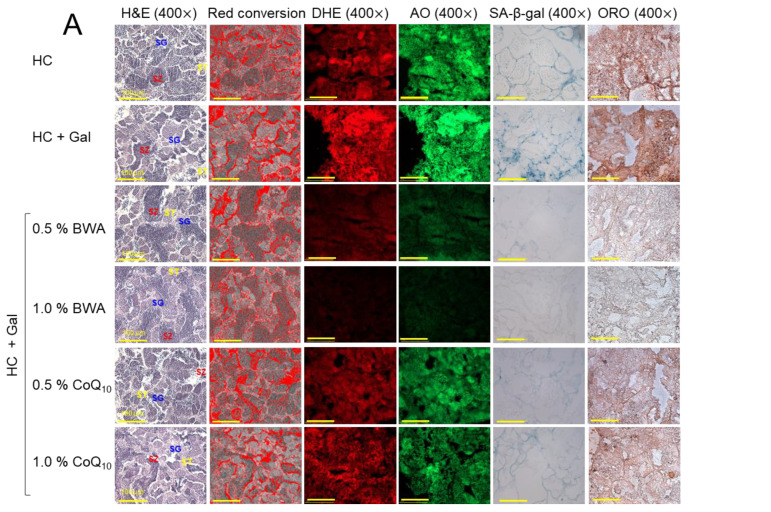

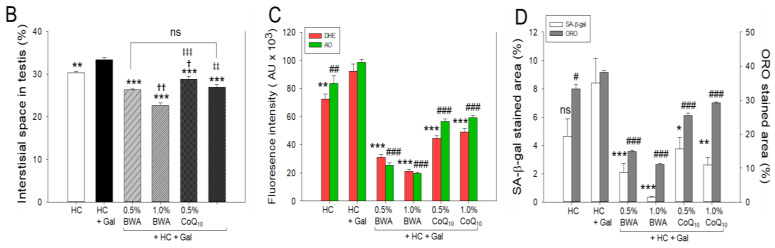
A comparative effect of beeswax alcohol (BWA) and coenzyme Q_10_ (CoQ_10_) on the testicular tissue of zebrafish fed with a high-cholesterol (HC, final 4%, *w/w*) and galactose (Gal, final 10%, *w/w*) diet. (**A**) Hematoxylin and eosin (H&E) staining; Image J-based interchange of white color (depicting the interstitial space) to red color (red conversion) at the white color threshold value (20–120) to enhance the clarity; and SG, ST, and SZ abbreviated for spermatogonia, spermatocyte, and spermatozoa. Dihydroethidium (DHE) and acridine orange (AO) staining indicate the generation of reactive oxygen species (ROS) and apoptosis. Oil red O (ORO) and senescent-associated β-galactosidase (SA-β-gal) staining. [100 μm, scale bar]. (**B**) Quantification of interstitial space in the testis section. (**C**) DHE and AO fluorescence intensity quantification employing Image J software. (**D**) Estimation of SA-β-gal and ORO-stained areas across the different groups. The statistical divergence between the groups was denoted by * (*p* < 0.05), ** (*p* < 0.01), and *** (*p* < 0.001) for HC+Gal (for interstitial space, DHE fluorescent intensity, and SA-β-gal-stained area) while ^#^ (*p* < 0.05), ^##^ (*p* < 0.01), and ^###^ (*p* < 0.001) were for HC+Gal (for AO fluorescent intensity and ORO-stained area). ^†^ (*p* < 0.05) and ^††^ (*p* < 0.01) were for the HC+Gal+0.5% BWA group while ^‡‡^ (*p* < 0.01) and ^‡‡‡^ (*p* < 0.001) were for the HC+Gal+1.0% BWA group; ns represents a non-significant difference between the groups.

The testis histology evaluated by H&E staining revealed a substantial adverse effect of HC on the testis that was further worsened in the presence of Gal. The HC+Gal group noticed a haphazard tubular structure with vague spermatocyte (ST) and spermatozoa (SZ) arrangement with dilatated interstitial space between the seminiferous tubules. The HC+Gal-induced testis damage was substantially prevented by the consumption of BWA and CoQ_10_, as manifested by the significant 12%, 32%, 14%, and 19% reduction in the interstitial space between the seminiferous tubules in response to 0.5% and 1.0% BWA and 0.5% CoQ_10_ and 1.0% CoQ_10_, respectively (Figure 9A,B).

The DHE and AO fluorescence imaging corresponding to ROS and apoptosis demonstrated the provocative effect of Gal to augment HC-induced ROS and apoptosis as documented by a significantly ~1.8-fold higher DHE and AO fluorescent intensity than the HC group (Figure 9A,C). The consumption of BWA at both 0.5% and 1.0% efficiently countered the HC+Gal-induced ROS production, as documented by a significantly 2.9-fold- and 4.3-fold-reduced DHE fluorescent intensity compared to the HC+Gal group. Likewise, a ~2-fold-reduced DHE-stained area was observed in both CoQ_10_ groups, 0.5% and 1.0%, compared to the HC+Gal group. The AO fluorescent staining revealed a substantial effect of BWA and CoQ_10_ in eliminating HC+Gal-induced apoptosis. However, when compared with CoQ_10_, BWA at 0.5% and 1.0% was found to be more effective in preventing apoptosis, marked by 2.2-fold and 3.1-fold, respectively, reductions in AO fluorescent intensity compared to CoQ_10_ at the respective concentrations.

A higher accumulation of lipids in response to HC+Gal was noticed in the testis, which was significantly prevented by consuming BWA and CoQ_10_ (Figure 9A,D). However, compared to CoQ_10_, BWA displayed much higher efficacy with 1.6-fold- and 2.6-fold-reduced ORO-stained areas in 0.5% and 1.0% BWA-consumed groups compared to CoQ_10_ at the respective concentrations. Similarly, both BWA and CoQ_10_ significantly altered the HC+Gal-induced cellular senescence; however, the higher efficacy of BWA compared to CoQ_10_ was noticed as depicted by significantly 1.8-fold- and 7.7-fold-reduced senescence-positive cells in response to 0.5% and 1.0% BWA than that of CoQ_10_ at 0.5% and 1.0%, respectively (Figure 9A,D).

## 3. Discussion

The composition of beeswax is substantially impacted by factors such as honeybee families and breed [1], the age of the wax, and varying climatic conditions [28], which also influence the quality of BWA. An earlier study documented the presence of fatty alcohols C33 and C35 in *A. mellifera* [29]. However, unlike this, we did not observe the presence of alcohols of C33 and C35 in used BWA, signifying that BWA composition substantially varied based on the honeybee types and source of beeswax. In contrast, the present findings are similar to the earlier study [28] documenting the presence of six distinct alcohols (C24, C26, C28, C30, C32, and C34) in the beeswax of *A. mellifera* L. with a high prevalence of C30 and C32 alcohols similar to the present study.

It is well known that a long-term HC diet, around 12~20 weeks, causes ruinous vascular lipid accumulation and damage to main organs, such as the liver, kidney, ovary, and testis with hypercholesterolemia and obesity in zebrafish [30,31]. Zebrafish were picked as a model organism in the present study owing to their significant genetic resemblance to humans [32], making them an excellent choice for preclinical research [33]. Of note, several key receptors and enzymes involved in lipid metabolism in zebrafish closely resemble those in humans [34], establishing them as a valuable model for investigating lipoprotein-related research.

In the current study, galactose (final 10%, *w/w*) combined with HC resulted in more rapid and severe damage in the major organs with rigorous inflammation, senescence, and atherogenic blood lipid profiles. After 12 weeks of consumption, the HC+Gal group showed the highest increase in body weight (Table 1), blood TC and TG, and blood AST/ALT (Figure 1, Figure 2 and Figure 3). Interestingly, the HC+Gal group revealed the lowest blood HDL-C and the highest extent of cell senescence, fatty liver change, neutrophil infiltration, and IL-6 production in the liver (Figure 4, Figure 5 and Figure 6). The HC+Gal group also exhibited the highest extent of ROS production, apoptosis, and senescence in the kidney, ovary, and testis (Figure 7, Figure 8 and Figure 9). These results make a good agreement with previous reports that the administration of D-galactose accelerated aging and reduced fertility with exacerbation of cognitive dysfunction and neurodegeneration [35,36].

However, both the 0.5% and 1.0% BWA group showed higher survivability, 96% and 100%, respectively, than the HC+Gal group, around 92%, while the 0.5% and 1.0% CoQ_10_ group showed lower survivability, around 92% and 89%, respectively than the HC+Gal group (Table 1 and Figure 1). BWA groups exhibited higher efficacy than CoQ_10_ groups in ameliorating the lipid profile and organ injury, the least inflammation, cellular senescence, apoptosis, and ROS production in the liver, kidney, ovary, and testis. Among the BWA-supplemented groups, the 1.0% BWA group showed remarkably superior behaviors than the 0.5% BWA in improving blood lipid profiles by significantly lowering TC, TG, TG/HDL-C, and LDL-C:HDL-C with raising HDL-C and HDL-C/TC (%). However, CoQ_10_ groups did not show a difference between 0.5% and 1.0% in blood lipid profiles; they exhibited an atherogenic lipid profile without lowering TG and raising HDL-C. In blood profiles and histological analysis of the liver, there was no difference in neutrophil infiltration, IL-6 production, and cellular senescence between the 0.5% and 1.0% CoQ_10_ groups, suggesting that CoQ_10_ supplementation did not protect the zebrafish from galactose toxicity in both dosages.

Although CoQ_10_ is known to protect cellular membranes against oxidative stress in mitochondria and extra-mitochondria as a lipid-soluble antioxidant via removing ROS, there might be different efficacy depending on the dosage and supplementation period. A meta-analysis with a human study showed that 400–500 mg/day of CoQ_10_ supplementation decreased the TC and TG levels with an increase in HDL-C [37]; however, other clinical trials showed that consumption of 150 mg/day of CoQ_10_ in patients with diabetes for 12 weeks resulted in no change in TC, with TG and HDL-C being decreased [38]. While several research studies showed conflicting results, and there has been no established ideal dose of CoQ_10_, it has been generally accepted that the recommended daily dose is 50 to 300 mg for adults to treat cardiovascular disease, dyslipidemia, and sarcopenia [39,40]. Recently, six-month supplementation with high-dose CoQ_10_, 240 mg/day, has been shown to improve liver steatosis in patients with metabolic dysfunction associated with steatosis liver disease [41].

However, there was no beneficial effect on the decrease in blood TC and TG and increase in HDL-C levels in the CoQ_10_ group, except for a decrease in LDL-C [41]. The accumulating finding showed the disparity of the CoQ_10_ functionality [41]. In the present study, in the 1.0% CoQ_10_ group, an intake of 0.2 mg of CoQ_10_ per day is equivalent to the 616 mg and 456 mg per day humanized dose (assumption of 60 kg body weight) calculated based on the average body weight of zebrafish at 370 mg (week 0) and 580 mg (week 12), respectively, employing the equation suggested for conversion of the estimated human equivalent dose [42]. Despite the high dose, we have noticed no effect of CoQ_10_ to alleviate the TG level; though, a substantial effect was observed to restore HC+Gal-disturbed TC and HDL-C levels. However, the effect was substantially inferior compared to the effect exerted by BWA. The 1.0% CoQ_10_ supplemented group was found to be ineffective in diminishing HC+Gal-induced cellular senescence and fatty liver changes, suggesting that there is not much of an effect of CoQ_10_ in preventing hepatic damage. The outcome is supported by the elevated level of important hepatic function biomarker AST in response to CoQ_10_ consumption in the presence of HC+Gal.

It has been well known that high-cholesterol or galactose supplementation induces oxidative stress to cause proinflammatory and senescence signaling. However, no study has investigated the physiological effect of the co-supplementation with cholesterol and galactose. As far as the authors’ knowledge, the current study is the first report to demonstrate that the co-supplementation of cholesterol and galactose induced more severe detrimental effects than the adverse effect posed by HC alone. Also, it is the first report deciphering a comparative effect of BWA and CoQ_10_ against high-cholesterol- and galactose-induced adversity.

The higher efficacy of BWA over CoQ_10_ to prevent HC+Gal-induced damage is probably due to the higher potency of BWA as an antioxidant, which efficiently manages the oxidative stress that has been recognized as the major culprit in inducing apoptosis [43,44] and cellular senescence [45,46]. This notion is supported by the earlier report signifying the higher antioxidant efficacy of BWA than CoQ_10_ [12] that leads to the protection of LDL oxidation and prevention of ROS generation against the CML-posed toxicity and consequently rescues the zebrafish embryos from the apoptotic cell death [12,13]. Also, the impact of BWA consumption on preventing lipid peroxidation oxidative damage of gastric mucosa of rats strengthens the present findings [5,11,47]. Even more, in the clinical study, BWA (100 mg/day) consumption improved the antioxidant status of middle-aged and older human subjects [12].

## 4. Materials and Methods

### 4.1. Materials

A block of beeswax from *Apis mellifera* (mainly mellifera linage) was used as a source material to extract beeswax alcohol (BWA). The BWA was extracted from beeswax using saponification and repeated organic solvent extraction at the National Center for Scientific Research (CNIC), Havana, Cuba. In brief, the beeswax was melted at shaking conditions in the presence of potassium hydroxide (KOH) for saponification. The saponified beeswax was processed for purification using organic solvents (acetone and hexane). Subsequently, the organic phase was evaporated, and the dried final product was processed for milling (stainless steel blade miller). The milled product was homogenized to obtain BWA. A schematic representation of the BWA extraction process is provided as Supplementary Figure S1. The CNIC-sourced BWA was examined for its purity and chemical composition of long-chain aliphatic alcohols (LCAAs) by Raydel^®^ Australia Pty, Ltd. (Thornleigh, NSW, Australia). The quality analysis of BWA revealed ≥ 86% purity and the presence of six long-chain aliphatic alcohols (LCAAs) namely tetracosanol (6.1%), hexacosanol (10.7%), octacosanol (13.8%), triacontanol (30.5%), dotriacontanol (22.1%), and tetratriacontanol (2.9%). A certificate of analysis with the detailed chemical composition of the used BWA is provided in Supplementary Table S1. The CoQ_10_ was purchased from Sigma-Aldrich (>98% HPLC, Cat No. 303-98-0, St. Louis, MO, USA). All the other chemicals and reagents used in this study were of analytical grade and used as supplied. Supplementary Material S1 depicts the specifications of the used chemicals.

### 4.2. Zebrafish Aquaculture

An aerated tank with a constant water supply was used to maintain the zebrafish. The atmospheric conditions were maintained at 27 ± 1 °C with 14 h light and 10 h dark cycles. Zebrafish were fed with normal tetrabit (ND, Gmbh D49304, Melle, Germany) following the standard protocols adopted by the Animal Care Committee and the Use of Raydel Research Institute (approval code RRI-23-007; approval date 27 July 2023).

### 4.3. Preparation of High-Cholesterol Diet Infused with BWA or CoQ_10_

The normal tetrabit (ND) was blended with cholesterol (final 4%, *w/w*) to make the high-cholesterol (HC) diet [48]. The HC diet was further mixed with galactose (Gal, final 10%, *w/w*) to make HC supplemented with galactose (HC+Gal). HC+Gal was further mixed with BWA at 0.5% or 1.0% (*w/w*) to make two distinct BWA-infused diets named HC+Gal with 0.5% BWA and HC+Gal with 1.0% BWA, respectively. Similarly, two distinct CoQ_10_-supplemented diets were prepared by mixing HC+Gal with CoQ_10_ at 0.5% or 1.0% (*w/w*) and designated as HC+Gal with 0.5% CoQ_10_ and HC+Gal with 1.0% CoQ_10_, respectively. Table 2 depicts the specific amount of ND, cholesterol, galactose, BWA, and CoQ_10_ to prepare different diets for zebrafish feeding. The specified diet, 10 mg/zebrafish (twice a day), was provided during the 12 weeks of the experimental period. The selection of BWA dosages (final 0.5% and 1.0%, *w/w*) was based on the preliminary experiments where we tested different dosages of BWA (final 0.1% and 10%, *w/w*) in hyperlipidemic zebrafish and observed their impact on blood lipid profile and hepatic function biomarkers (AST and ALT). The results indicated significant changes in plasma lipid profiles and AST and ALT levels at BWA concentrations between 0.5% (*w/w*) and 1.0% (*w/w*), while no significant difference was observed at concentrations exceeding 1.0% (*w/w*) up to 10% (*w/w*). Consequently, we chose 1.0% (*w/w*) BWA as the highest dose for the present study. For the comparative study (between BWA and CoQ_10_), we used a similar dose of 1.0% (*w/w*) CoQ_10_.

### 4.4. Zebrafish Fed with Different Diets

**Figure 10:**
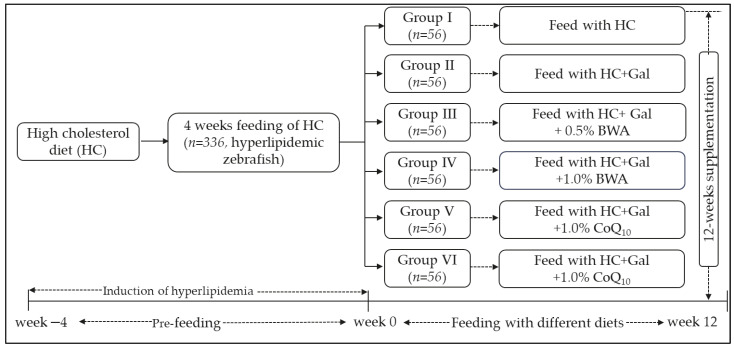
A schematic representation of the study plan. HC represents the high-cholesterol diet (final 4%, *w/w*) and Gal represents galactose (final 10%, *w/w*), while HC+Gal+BWA 0.5% and 1.0% represent HC+Gal supplemented with beeswax alcohol at 0.5% and 1.0% (final, *w/w*), and HC+Gal+CoQ_10_ 0.5% and 1.0% represent HC+Gal supplemented with coenzyme Q_10_ (CoQ_10_) at 0.5% and 1.0% (final, *w/w*).

Adult zebrafish (*n* = 336) were fed with HC for 4 weeks prior to supplementation with different diets (Figure 10). The 4 weeks of supplementation of HC was performed to induce hyperlipidemia in zebrafish before introducing different dietary treatments [48]. The HC-supplemented zebrafish (4 weeks) were randomly allocated into six separate groups (*n* = 56 for each group) and fed with the specified diets for the 12 weeks. Zebrafish in groups I and II were fed with only HC and HC+Gal, respectively. Zebrafish in groups III and IV were maintained on the HC+Gal with 0.5% BWA and HC+Gal with 1.0% BWA diets, respectively. Likewise, the group V and VI zebrafish were nourished with HC+Gal with 0.5% CoQ_10_ and HC+Gal with 1.0% CoQ_10_, respectively (Figure 10). During the 12 weeks of supplementation, the survivability and body weight of the zebrafish (Group I-VI) were monitored periodically.

### 4.5. Blood and Tissue Collection

After 12 weeks, zebrafish in each group were sacrificed by hypothermal shock [48], and immediately, blood was collected from puncturing the heart. The blood was collected in tubes pre-washed with ethylenediaminetetraacetic acid (EDTA). The different organs (liver, kidney, testis, and ovaries) were surgically extracted and preserved in 10% formalin for further use.

### 4.6. Plasma Analysis

The collected blood was centrifuged to obtain plasma, which was processed for the quantification of total cholesterol (TC), triglycerides (TGs), high-density lipoprotein cholesterol (HDL-C), aspartate aminotransferase (AST), and alanine aminotransferase (ALT) using commercial kits following the manufacturers’ suggested method. A detailed methodology of plasma analysis is described in Supplementary Material S2.

### 4.7. Histologic Analysis

The tissue (liver, kidney, testis, and ovary) was preserved and fixed in 10% formalin (Section 2.4), further dehydrated by treatment with a series of alcohols, and subsequently fixed in paraffin. A 7 μm section of the tissue embedded in the paraffin block was obtained using a microtome. The respective tissue sections were processed for Hematoxylin and eosin (H&E) staining to determine histological changes following the earlier described method [49].

The lipid accumulation in the tissue was examined by Oil red O (ORO) staining [48]. In brief, organ sections (5 μm) were pooled with ORO stain and subsequently incubated at 60 °C for 5 min. The excess ORO was rinsed, subsequently stained with hematoxylin for 30 s, and visualized under microscopy.

### 4.8. Immunohistochemical (IHC) Staining

The hepatic interleukin (IL)-6 level was examined by IHC analysis [50]. The tissue section was covered with IL-6-specified 200× diluted primary immunoglobulin (ab9324, Abcam, London, UK) followed by 16 hr incubation at 4 °C. The IL-6-complexed immunoglobulins were detected by applying 1000× diluted anti-IL-6 antibodies (enzyme-linked), and the section was developed using the chromogenic substrate utilizing the EnVison + system-HRP polymer kit (Dako, Glostrup, Denmark).

### 4.9. Dihydroethidium (DHE) and Acridine Orange (AO) Staining

The dihydroethidium (DHE) [51] and acridine orange (AO) [52] staining was performed by adding 200 μL of DHE (30 μM) and AO (5 μg/mL) solution to the tissue section. After 5 min incubation in the dark, the stained section was rinsed and analyzed under fluorescent microscopy operated at the 585 nm (excitation) and 615 nm (emission) wavelengths for DHE fluorescence and 505 nm (excitation) and 535 nm (emission) wavelengths for AO fluorescence.

### 4.10. Senescence Staining

Senescence was detected by a senescent-associated β-galactosidase (SA-β-gal) assay [53]. Briefly, the tissue section was treated with paraformaldehyde (4%) for 5 min, and subsequently, 0.1% of X-gal (5-bromo-4-chloro-3-indolyl-β-D-galactopyranoside) was added. After 14 hr, the section was washed with phosphate-buffered saline (PBS) and visualized under the microscope.

### 4.11. Statistical Analysis

Mean ± SEM of the triplicate values obtained from the experimental data was used to analyze the statistical divergence between the groups following One-Way Analysis of Variance (ANOVA)–Tukey’s post hoc analysis using Statistical Package for the Social Sciences platform (SPSS, Inc., Chicago, IL, USA).

## 5. Conclusions

A comparable 12-week consumption study of BWA and CoQ_10_ disclosed the higher efficacy of BWA over CoQ_10_ in preventing adversity posed by the dietary consumption of HC+Gal. BWA, precisely at 1.0%, substantially prevents HC+Gal-provoked dyslipidemia by alleviating TC and TG levels and simultaneously increasing the HDL-C level. BWA also restricts HC+Gal-induced oxidative stress and inflammation, averts hepatic cellular senescence and fatty liver changes, and improves hepatic functionality. Consistently, BWA consumption was found effective in abolishing HC+Gal-invoked impairment of kidneys and reproductive organs. The findings underscore BWA’s superior functionality over CoQ_10_ in mitigating HC+Gal-induced alterations, making BWA a viable nutraceutical for managing cholesterol- and galactose-related concerns.

## Figures and Tables

**Table 1:** Survivability and body weight of zebrafish across the different groups at the beginning (week 0) and at 12 weeks of consumption of the respective diets.

Survivability and BW of Zebrafish	HC	HC+Gal	HC+Gal +0.5% BWA	HC+Gal +1.0% BWA	HC+Gal +0.5% CoQ_10_	HC+Gal +1.0% CoQ_10_
Week 0 (n)	56	56	56	56	56	56
Week 12 (n)	54	52	54	56	52	50
Survivability	96.4	92.9	96.4	100.0	92.9	89.3
BW at week 0	366.4 ± 24.5	368.3 ± 22.9	372.3 ± 20.1	365.1 ± 17.9	378 ± 21.5	372.3 ± 22.3
BW at week 12	590.9 ± 31.8	614.3 ± 45.3	558.7 ± 37.4	564.5 ± 36.8	561.7 ± 37.0	565.9 ± 45.0
Net increase in BW (mg)	224.5 ± 8.7	246.1 ± 25.4	180.1 ± 19.8	212.4 ± 20	175.5 ± 16.2	164.7 ± 27
Net increase in BW (%)	161.3 ± 2.3	166.8 ± 3.9	150.1 ± 3.9	154.6 ± 3.0	148.6 ± 2.1	152.0 ± 4.7

BW, body weight; BWA, beeswax alcohol; CoQ_10_, coenzymeQ_10_; Gal, galactose; HC, high cholesterol.

**Table 2:** Composition of the six different diets prepared by cholesterol, galactose, beeswax alcohol, and coenzyme Q_10_.

Diet (10 mg)	HC	HC + Gal	HC+Gal + 0.5% BWA	HC+Gal + 1.0% BWA	HC+Gal + 0.5% CoQ_10_	HC+Gal + 1.0% CoQ_10_
Tetrabits	9.6	8.6	8.55	8.5	8.55	8.5
Galactose	0	1.0	1.0	1.0	1.0	1.0
Cholesterol	0.4	0.4	0.4	0.4	0.4	0.4
BWA	0	0	0.05	0.1	0	0
CoQ_10_	0	0	0	0	0.05	0.1
Total (mg)	10.0	10.0	10.0	10.0	10.0	10.0

BW, body weight; BWA, beeswax alcohol; CoQ_10_, coenzymeQ_10_; Gal, galactose; HC, high cholesterol.

## Data Availability

Data is contained within the article and Supplementary Materials.

## Supplementary Materials

The following supporting information can be downloaded at https://www.mdpi.com/article/10.3390/ph17091250/s1: Supplementary Figure S1: Sequential steps for the extraction of beeswax alcohol (BWA) from the beeswax. Supplementary Table S1: Certificate of analysis and composition of beeswax alcohol (BWA). Supplementary Material S1: List of the used chemicals. Supplementary material S2: A detailed methodology for the quantification of plasma lipid profiles and hepatic function biomarkers.

## References

[1] Fratini F., Cilia G., Turchi B., Felicioli A. (2016). Beeswax: A minireview of its antimicrobial activity and its application in medicine. Asian Pacif. J. Tropic. Med..

[2] Bradbear N. (2009). Bees and Their Role in Forest Livelihoods: A Guide to the Services Provided by Bees and the Sustainable Harvesting, Processing and Marketing of Their Products.

[3] Svečnjak L., Chesson L.A., Gallina A., Maia M., Martinello M., Mutinelli F., Muz M.N., Nunes F.M., Saucy F., Tipple B.J. (2019). Standard methods for *Apis mellifera* beeswax research. J. Apic. Res..

[4] Srisaipet A., Phromchan S., Jaipaeng T. (2017). Policosanol extraction from beeswax and improvement of the purity. MATEC Web Conf..

[5] Molina V., Mas R., Carbajal D. (2015). D-002 (beeswax alcohols): Concurrent joint health benefits and gastroprotection. Indian J. Pharm. Sci..

[6] Menéndez R., Amor A., González R., Jiménez S., Mas R. (2000). Inhibition of rat microsomal lipid peroxidation by the oral administration of D002. Braz. J. Med. Biol. Res..

[7] Lopez E., Illnait J., Molina V., Oyarzabal A., Fernandez L., Perez Y., Mas R., Mesa M., Fernandez J., Mendoza S. (2008). Effects of D-002 (beeswax alcohols) on lipid peroxidation in middle-aged and older subjects. Lat. Am. J. Pharm..

[8] Rodriguez I., Illnait J., Molina V., Oyarzabal A., Fernandez L., Mesa M., Más R., Mendoza S., Gámez R., Jiménez S. (2010). Comparison of the antioxidant effects of D-002 (beeswax alcohols) and grape seed extract (GSE) on plasma oxidative variables in healthy subjects. Lat. Am. J. Pharm..

[9] Menéndez R., Más R., Illnait J., Pérez J., Amor A.M., Fernández J.C., González R.M. (2001). Effects of D-002 on lipid peroxidation in older subjects. J. Med. Food.

[10] Illnait J., Rodríguez I., Mendoza S., Fernández Y., Mas R., Miranda M., Piñera J., Fernández J.C., Mesa M., Fernández L. (2013). Effects of D-002, a mixture of high molecular weight beeswax alcohols, on patients with nonalcoholic fatty liver disease. Korean J. Intern. Med..

[11] Pérez Y., Oyárzabal A., Mas R., Molina V., Jiménez S. (2013). Protective effect of D-002, a mixture of beeswax alcohols, against indomethacin-induced gastric ulcers and mechanism of action. J. Nat. Med..

[12] Cho K.-H., Baek S.-H., Nam H.-S., Bahuguna A., López-González L.E., Rodríguez-Cortina I., Illnait-Ferrer J., Fernández-Travieso J.C., Molina-Cuevas V., Pérez-Guerra Y. (2024). Beeswax alcohol prevents low-density lipoprotein oxidation and demonstrates antioxidant activities in zebrafish embryos and human subjects: A clinical study. Curr. Issues Mol. Biol..

[13] Cho K.-H., Baek S.-H., Nam H.-S., Bahuguna A. (2023). Enhancement of antioxidant and anti-glycation properties of beeswax alcohol in reconstituted high-density lipoprotein: Safeguarding against carboxymethyllysine toxicity in zebrafish. Antioxidants.

[14] Puig M.N., Castaño S.M., Ferreiro R.M., Clara M.V., Hernansez N.M. (2016). Effects of oral administration of D-002 (Beeswax alcohols) on histological and functional outcomes in a Rat model of antigen-induced arthritis: Preliminary Study. Int. J. Pharmacol. Phytochem. Ethnomed..

[15] Illnait J., Rodriguez I., Molina V., Mendoza S., Mas R., Fernandez L., Oyarzabal A., Perez Y., Mesa M., Fernández J. (2013). Effects of D-002 (beeswax alcohols) on gastrointestinal symptoms and oxidative markers in middle-aged and older subjects. Lat. Am. J. Pharm..

[16] Korean Food and Drug Administration (KFDA). https://www.foodsafetykorea.go.kr/portal/healthyfoodlife/searchHomeHFDetail.do?prdlstReportLedgNo=2023021000330706.

[17] Ardekani A., Tabrizi R., Maleki E., Bagheri Lankarani K., Heydari S.T., Moradinazar M., Akbari M. (2023). Effects of coenzyme Q_10_ supplementation on lipid profiles and liver enzymes of nonalcoholic fatty liver disease (NAFLD) patients: A systematic review and meta-analysis of randomized controlled trials. Food Sci. Nutr..

[18] Ashkani Esfahani S., Esmaeilzadeh E., Bagheri F., Emami Y., Farjam M. (2013). The effect of co-enzyme q10 on acute liver damage in rats, a biochemical and pathological study. Hepat. Mon..

[19] Jiménez-Santos M.A., Juárez-Rojop I.E., Tovilla-Zárate C.A., Espinosa-García M.T., Juárez-Oropeza M.A., Ramón-Frías T., Bermúdez-Ocaña D.Y., Díaz-Zagoya J.C. (2014). Coenzyme Q10 supplementation improves metabolic parameters, liver function and mitochondrial respiration in rats with high doses of atorvastatin and a cholesterol-rich diet. Lipids Health Dis..

[20] Pasha R., Moon T.W. (2017). Coenzyme Q_10_ protects against statin-induced myotoxicity in zebrafish larvae (*Danio rerio*). Environ. Toxicol. Pharmacol..

[21] Jaskelioff M., Muller F.L., Paik J.-H., Thomas E., Jiang S., Adams A.C., Sahin E., Kost-Alimova M., Protopopov A., Cadiñanos J. (2011). Telomerase reactivation reverses tissue degeneration in aged telomerase-deficient mice. Nature.

[22] Azman K.F., Zakaria R. (2019). D-Galactose-induced accelerated aging model: An overview. Biogerontology.

[23] Kumar H., Bhardwaj K., Valko M., Alomar S.Y., Alwasel S.H., Cruz-Martins N., Dhanjal D.S., Singh R., Kuča K., Verma R. (2022). Antioxidative potential of *Lactobacillus* sp. in ameliorating D-galactose-induced aging. Appl. Microbiol. Biotechnol..

[24] Azman K.F., Safdar A., Zakaria R. (2021). D-galactose-induced liver aging model: Its underlying mechanisms and potential therapeutic interventions. Exp. Gerontol..

[25] Du Z., Yang Y., Hu Y., Sun Y., Zhang S., Peng W., Zhong Y., Huang X., Kong W. (2012). A long-term high-fat diet increases oxidative stress, mitochondrial damage and apoptosis in the inner ear of D-galactose-induced aging rats. Hear. Res..

[26] Cui X., Zuo P., Zhang Q., Li X., Hu Y., Long J., Packer L., Liu J. (2006). Chronic systemic D-galactose exposure induces memory loss, neurodegeneration, and oxidative damage in mice: Protective effects of R-α-lipoic acid. J. Neurosci. Res..

[27] Han Y., Zee S., Cho K.-H. (2023). Beeswax alcohol and fermented black rice bran synergistically ameliorated hepatic injury and dyslipidemia to exert antioxidant and anti-inflammatory activity in ethanol-supplemented zebrafish. Biomolecules.

[28] Bonvehí J.S., Bermejo F.O. (2012). Detection of adulterated commercial Spanish beeswax. Food Chem..

[29] Aichholz R., Lorbeer E. (1999). Investigation of combwax of honeybees with high-temperature gas chromatography and high-temperature gas chromatography–chemical ionization mass spectrometry: I. High-temperature gas chromatography. J. Chromatogr. A.

[30] Cho K.-H., Kim J.-E., Nam H.-S., Baek S.-H., Bahuguna A. (2023). Consumption of policosanol (Raydel^®^) improves hepatic, renal, and reproductive functions in zebrafish: In vivo comparison study among Cuban, Chinese, and American policosanol. Pharmaceuticals.

[31] Yoon Y., Yoon J., Jang M.Y., Na Y., Ko Y., Choi J.H., Seok S.H. (2013). High cholesterol diet induces IL-1beta expression in adult but not larval zebrafish. PLoS ONE.

[32] Patton E.E., Zon L.I., Langenau D.M. (2021). Zebrafish disease models in drug discovery: From preclinical modelling to clinical trials. Nat. Rev. Drug Discov..

[33] Juan-García A., Bind M.-A., Engert F. (2020). Larval zebrafish as an in vitro model for evaluating toxicological effects of mycotoxins. Ecotoxicol. Environ. Saf..

[34] Fang L., Liu C., Miller Y.I. (2014). Zebrafish models of dyslipidemia: Relevance to atherosclerosis and angiogenesis. Transl. Res..

[35] Shen Y.X., Xu S.Y., Wei W., Sun X.X., Yang J., Liu L.H., Dong C. (2002). Melatonin reduces memory changes and neural oxidative damage in mice treated with D-galactose. J. Pineal Res..

[36] Liao C.H., Chen B.H., Chiang H.S., Chen C.W., Chen M.F., Ke C.C., Wang Y.Y., Lin W.N., Wang C.C., Lin Y.H. (2016). Optimizing a male reproductive aging mouse model by D-galactose injection. Int. J. Mol. Sci..

[37] Liu Z., Tian Z., Zhao D., Liang Y., Dai S., Liu M., Hou S., Dong X., Zhaxinima, Yang Y. (2023). Effects of coenzyme Q_10_ supplementation on lipid profiles in adults: A meta-analysis of randomized controlled trials. J. Clin. Endocrinol. Metab..

[38] Zahedi H., Eghtesadi S., Seifirad S., Rezaee N., Shidfar F., Heydari I., Golestan B., Jazayeri S. (2014). Effects of CoQ_10_ supplementation on lipid profiles and glycemic control in patients with Type 2 diabetes: A randomized, double blind, placebo-controlled trial. J. Diabetes Metab. Disord..

[39] Bolt J., Sandhu S., Mohammadi A. (2023). Effect of coenzyme Q_10_ supplementation on sarcopenia, frailty, and falls: A scoping review. J. Nutr. Health Aging.

[40] Hou S., Tian Z., Zhao D., Liang Y., Dai S., Ji Q., Fan Z., Liu Z., Liu M., Yang Y. (2023). Efficacy and optimal dose of coenzyme Q_10_ supplementation on inflammation-related biomarkers: AGRADE-assessed systematic review and updated meta-analysis of randomized controlled trials. Mol. Nutr. Food Res..

[41] Soleimani Damaneh M., Fatahi S., Aryaeian N., Bavi Behbahani H. (2023). The effect of coenzyme Q10 supplementation on liver enzymes: A systematic review and meta-analysis of randomized clinical trials. Food Sci Nutr..

[42] Nair A.B., Jacob S. (2016). A simple practice guide for dose conversion between animals and human. J. Basic Clin. Pharm..

[43] Kannan K., Jain S.K. (2000). Oxidative stress and apoptosis. Pathophysiology.

[44] Lee Y.-J., Cho H.-N., Soh J.-W., Jhon G.J., Cho C.-K., Chung H.-Y., Bae S., Lee S.-J., Lee Y.-S. (2003). Oxidative stress-induced apoptosis is mediated by ERK1/2 phosphorylation. Exp. Cell Res..

[45] Nousis L., Kanavaros P., Barbouti A. (2023). Oxidative stress-induced cellular senescence: Is labile iron the connecting link?. Antioxidants.

[46] Faraonio R. (2022). Oxidative stress and cell senescence process. Antioxidants.

[47] Molina V., Valdés S., Carbajal D., Arruzazabala L., Menéndez R., Más R. (2001). Antioxidant effect of D-002 on gastric mucosa of rats with experimentally induced injury. J. Med. Food.

[48] Cho K.-H., Kim J.-E., Bahuguna A., Kang D.-J. (2023). Long-term supplementation of ozonated sunflower oil improves dyslipidemia and hepatic inflammation in hyperlipidemic zebrafish: Suppression of oxidative stress and inflammation against carboxymethyllysine toxicity. Antioxidants.

[49] Fischer A.H., Jacobson K.A., Rose J., Zeller R. (2008). Hematoxylin and eosin staining of tissue and cell sections. Cold Spring Harb. Protoc..

[50] Al-Ghamdi T.H., Atta I.S. (2020). Efficacy of interleukin-6 in the induction of liver cell proliferation after hemi-hepatectomy: Histopathologic and immunohistochemical study. Int. J. Clin. Exp. Pathol..

[51] Owusu-Ansah E., Yavari A., Mandal S., Banerjee U. (2008). Distinct mitochondrial retrograde signals control the G1-S cell cycle checkpoint. Nat. Genet..

[52] Umali J., Hawkey-Noble A., French C.R. (2019). Loss of foxc1 in zebrafish reduces optic nerve size and cell number in the retinal ganglion cell layer. Vis. Res..

[53] Cho K.-H., Bahuguna A., Kang D.-J., Kim J.-E. (2024). Prolonged supplementation of ozonated sunflower oil bestows an antiaging effect, improves blood lipid profile and spinal deformities, and protects vital organs of zebrafish (*Danio rerio*) against age-related degeneration: Two-years consumption study. Antioxidants.

